# Transmission of a novel sonotubometry acoustic click stimulus in healthy and patulous eustachian tube subjects: a retrospective case -control study

**DOI:** 10.1186/s40463-017-0227-x

**Published:** 2017-06-15

**Authors:** Justin M. Pyne, Yaw Amoako-Tuffour, Guy Earle, Graham McIntyre, Michael B. Butler, Manohar Bance

**Affiliations:** 1 Faculty of Medicine, Dalhousie University, Halifax, NS Canada; 2 Department of Surgery, Division of Otolaryngology – Head and Neck Surgery, Faculty of Medicine, Dalhousie University, Halifax, NS Canada

**Keywords:** Eustachian tube, Patulous, Sonotubometry, Clicks

## Abstract

**Background:**

Eustachian tube (ET) dysfunction can be very difficult to diagnose accurately. Our aim is to determine whether a newly developed sonotubometric test using clicks can reliably detect ET opening during swallowing in normal ET subjects, and patulous ET (PET) in subjects with ET dysfunction.

**Methods:**

Sixteen subjects (19 normal ET ears and 6 PET ears) were individually placed in a sound-isolated audiometry booth and subjected to a 1000Hz click train stimulus, played through the nose. PET subjects were identified through the ET clinic at our institution, while healthy subjects were recruited. Transmission through the ET was recorded by a microphone in the ear ipsilateral to the presenting nostril, during no swallow and swallow states, and this was used to compute a power ratio (power in the frequency range of interest to the whole frequency range). The power transmission ratio both before and after the swallow was averaged, and represented the baseline (BaseR). The power transmission ratio during swallow represented the peak (PeakR). The same process was repeated in the absence of a stimulus to account for swallowing noise. Wilcoxon rank rum tests were performed to determine statistical significance.

**Results:**

It was found that for healthy ET patients, the median difference between the PeakR and BaseR was 0.51 (*p* = 0.004). For the PET patients in this study, the median difference between the PeakR and the BaseR was 3.30 (*p* = 0.041). Comparing the baseline between groups revealed that PET patients had a median BaseR 1.05 higher than healthy ET patients. PET patients had a median PeakR of 3.84 higher than healthy ET patients. Both were deemed to be statistically significant (*p* = 0.003, *p* = 0.003 respectively). A significant difference was found between median PeakR for the stimulus and no-stimulus condition for the healthy ET group (0.59, *p* < 0.001) and for the PET group (4.39, *p* = 0.031), indicating that it was unlikely that swallowing noise caused false positive results.

**Conclusion:**

The results of this study suggest that a novel click stimulus is capable of detecting ET opening during swallowing in healthy patients as well as highlighting PET in diseased subjects.

## Background

In PET, first described by H. Schwartze in 1864, the ET stays open intermittently, or sometimes persistently [1]. This can cause symptoms of autophony, the sensation of hearing one’s own voice loudly, aerophony, hearing one’s own respiratory sounds, as well as aural fullness [2].

The symptoms of PET, such as autophony can overlap with other disorders, such as superior canal dehiscence. In the classic history of PET, autophony and aerophony are made worse by standing and exercise, and improved by lying down [3]. However, symptoms can be intermittent and not present when the patient is seen in clinic. The definitive diagnosis is made if the eardrum is seen to move with respiration, which can be exacerbated by having the subject sit upright and take deep breaths while occluding one nostril, to accentuate nasopharyngeal pressure changes [3].

Several different methods have been employed over the years to attempt to evaluate ET function. In 1981, Bluestone and Cantekin proposed the nine-step inflation/deflation test for testing opening during swallowing, particularly to test if an ET was hypofunctioning [4]. For PET, various authors have described measuring aspects of middle ear compliance continuously using acoustic immittance probes, while the subject forcefully breathes. This can detect movement of the eardrum synchronous with respiration [4–7]. However, this is subject to artefact from the noise of breathing, and is only positive if the patient’s ET is truly patent exactly at the time of testing.

A promising and well-described tool to test ET function, in relatively physiologic conditions, is sonotubometry. This measures sound intensity at the ear when a stimulus is introduced into the nasal cavity, and looks for changes in this intensity associated with ET opening. While sonotubometry is a relatively non-invasive means of evaluating ET function, one of the biggest pitfalls of this method is the potential for interference caused by biological noise sources such as swallowing, saliva movement, and breathing, all of which can affect the sound pressure level (SPL) in the nasopharynx, and thus the MEC. Modern sonotubometry began when in 1978, Virtanen described an experimental setup that involved placing a sound source near one nostril and a placing a microphone in the ipsilateral EAC [8]. A stimulus was then played, and the transfer function between the naris and ear canal was recorded. On the basis that most pharyngeal movement caused noise interference at 5 KHz and below, it was suggested that stimuli of 6 KHz or above are most favourable for ET evaluation [9]. Recently the perfect sequences (PSEQ) broadband stimulus has been described for use in the sonotubometry method. It has been reported that this stimulus is able to detect ET opening in both healthy and diseased states more reliably than pure tone testing alone [10–12]. However this method of testing requires customized software and implementation design, equipment that is not readily available to audiologists.

Despite the prevalent use of sonotubometry in the evaluation of ET function, little evidence exists on the implementation of novel stimuli in this technique, and very little for testing specifically for PET. Our experience is that PET patients who have been witnessed to have eardrum movements with respiration will still commonly continue to describe symptoms of autophony and aerophony even when the eardrum stops moving when examined with microscopy or with immitance testing, as this phenomenon is often intermittent. Hence, we would like to develop a test that can directly measure reduced acoustic impedance for sound from the nasopharynx to the MEC.

The aim of this study is to determine whether a novel click train stimulus is capable of detecting ET opening during swallowing in healthy patients as well as characterizing PET in affected subjects. The click train is a sequence of square pulses that produce a broadband signal in the frequency domain. So, in theory, more information about the state of the ET should be derived by using a broader band of test frequencies than the single tone more commonly used, and hence increase our ability to differentiate between healthy and diseased states. As the clicks are similar to those used in auditory brainstem response (ABR) testing, they can be provided by any ABR machine and thus clinicians (audiologists, otologists, etc.) would readily have access to this equipment. Additionally, because clicks are a shorter stimulus than PSEQ stimuli, they could potentially track a rapidly dynamically changing system with higher temporal resolution than PSEQ. As PET subjects are suspected to have more patent ETs than healthy ET subjects, we expect that that the PET group will display only a modest change in acoustic power conduction to the EAC during swallowing compared to baseline no swallow testing, as the ET is open in both conditions. Conversely, the healthy group should yield a greater change in acoustic transmission during swallowing, indicating a transition state from a closed to an open condition has occurred.

## Methods

### Subject selection

Our study included 19 ears from 11 healthy ET subjects (normals) and 6 ears from 5 PET patients. This study was approved by our institutional research ethics board. Subjects were identified through the Eustachian Tube clinic at our institution as having suspected PET and asked to participate in this study. These subjects experienced one or more of the symptoms of autophony, aerophony, and aural fullness. All PET subjects were examined with otoscopy and microscopy to confirm that there was no obstruction in the ear canal, no current visible ear disease, and that the TM was moving with respiration prior to testing. Any subjects not meeting these criteria were excluded from the PET group. These were our confirmed PET subjects. One PET subject had both ears tested. Four PET subjects had only their suspected PET ear tested.

Healthy subjects, those with no ET dysfunction, were identified through the Otology clinic at the Queen Elizabeth II Health Sciences Center, and we also recruited healthy volunteers. Only ears with no prior history of disease, no previous ear surgery, and no current ET dysfunction were included in the healthy group. All healthy subjects were examined with otoscopic technique to confirm that there was no obstruction in the ear canal, no current visible ear disease, and that the TM was not moving with respiration prior to testing. Any healthy subjects not meeting these criteria were excluded. One ear was excluded due to the presence of a TM perforation and two ears were excluded due to prior history of middle ear disease.

### Data collection

A 1000 Hz click train stimulus and capture system was developed using LabVIEW (2013 version) and a Data Acquisition Module (DAQ, National Instruments - 6216). Each click comprised a rectangular signal of 200 μs duration that drove an Etymotic ER – 2 speaker (Etymotic Research Inc.) The DAQ sampled at a rate of 100 kHz. Experimental setup consisted of a desktop computer running LabVIEW, a DAQ, a button used to indicate swallowing by the subject, and an audiometer connected to the stimulus speaker. An ER-7C microphone, which was imbedded in a small foam insert, was placed in the external auditory canal of the ear of the subject of interest, an additional mic was placed in the contralateral naris, and the stimulus speaker embedded in a foam insert was placed in the ipsilateral naris. Both microphones were looped back through the DAQ and the data was collected on the desktop.

For each measurement, participants were seated in a sound attenuating audiometry booth, and all previously mentioned equipment was placed in its respective body aperture. Each participant was instructed to close his or her mouth. The operator initiated the 110 dB, 1000 Hz click stimulus from the desktop computer located outside of the room. The participant was then instructed to press down and continue to hold the indicator button, swallow, and release the button once finished swallowing. This process was repeated twice for each ear of interest, in both normal and PET subjects. As a control, the entire process was repeated for each ear in the absence of a stimulus.

Results were analyzed in MATLAB (2016a). For each click transmitted through the ET, the response in the external auditory canal (EAC) was recorded as a sequence of sound pressure level measurements. The Fast Fourier Transform was performed on these recorded responses to determine the power contributed from each frequency bin. Based on the results of a previous case study indicating range of frequencies that are most enhanced by ET opening, the spectral power contributions from the frequency range of 780–3125 Hz were divided by the spectral power contributions from all other frequencies below and above this band, up to 10 kHz [13]. In this sense, a power ratio was calculated for each click response that compared the power in the frequency band of interest, to the power outside of this band. The mean of these ratios outside of the swallowing period served as the baseline power transmission ratio (BaseR) for the subjects. The same ratio during the act of swallowing was simply reported as the peak power transmission ratio (PeakR). Together, these values reduced the measurement of a subject to two figures that facilitated a subject-to-subject comparison.

### Statistical analysis

For statistical analysis, the baseline and peak power transmission ratios were compared between healthy and PET subjects, as well as within each subject group. A case control design was implemented. with PET patients representing cases and healthy ET patients representing controls. Wilcoxon rank sum tests were performed to determine statistical significance. All analysis was performed with R version 3.3.1 (“Bug in Your Hair”). Effect sizes are presented as medians and approximate 95% confidence intervals. Non-parametric testing was utilized as sample sizes in our study were insufficient to rely on assumptions of asymptomatic normality for the purposes of hypothesis testing. A p value <0.05 was considered statistically significant (95% confidence interval.)

## Results

A total of 19 ears from 11 healthy ET subjects, and 6 ears from 5 PET subjects were subjected to sonotubometric testing using the novel 1000 Hz click stimulus under controlled conditions. The healthy subject group consisted of two females and nine males aged 21 to 69 (mean age =33.4 years), and the PET subject group consisted of 1 female and 4 males aged 51 to 84 (mean age =70 years). One male PET subject had both ears tested.

**Fig. 1:**
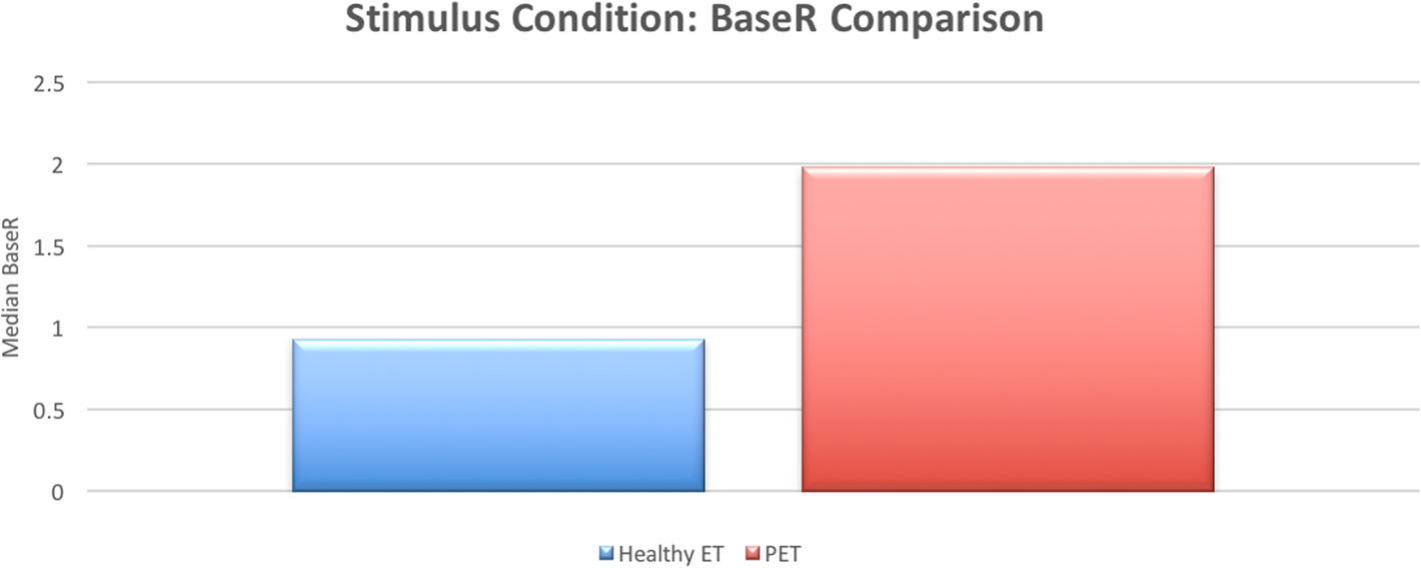
Comparison of BaseR between healthy ET and PET groups. A bar graph representation of the baseline power transmission ratio for the healthy ET and PET groups. The PET group (represented by the *red bar*) was found to have a significantly higher BaseR than the healthy ET group (represented by the *blue bar*). This finding was statistically significant (*p* = 0.003)

**Fig. 2:**
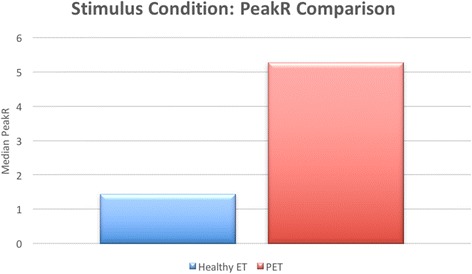
Comparison of PeakR between healthy ET and PET groups. A bar graph representation of the peak power transmission ratio for the healthy ET and PET groups. The PET group (represented by the *red bar*) was found to have a significantly higher PeakR than the healthy ET group (represented by the *blue bar*). This finding was statistically significant (*p* = 0.003)

For comparison between groups, the difference between median BaseR and median PeakR of each subject group was evaluated (Table 1). It was found that the difference between median BaseR for healthy ET and PET was 1.05, which was statistically significant (*p* = 0.003, Fig. 1). The median PeakR difference between groups was found to be 3.84, also statistically significant (*p* = 0.003, Fig. 2). The difference between median PeakR and median BaseR for the healthy ET group was 0.51, and 3.30 for the PET group. Both were found to be statistically significant (*p* = 0.004, *p* = 0.041, respectively, Table 2)

**Table 1:** Median baseline and peak power transmission ratios

Healthy ET		PET	
BaseR	PeakR	BaseR	PeakR
0.91	1.44	1.98	5.28

**Table 2:** Wilcoxon rank sum test for power transmission ratio comparison between healthy ET and PET groups

	Median difference	95% CI	p
Healthy ET BaseR vs. PET BaseR	1.05	(0.39 to 2.11)	0.003
Healthy ET PeakR vs. PET PeakR	3.84	(0.57 to 4.63)	0.003
Healthy ET BaseR vs. Healthy PeakR	0.51	(0.12 to 1.20)	0.004
PET BaseR vs. PET PeakR	3.30	(0.083 to 4.48)	0.041

To determine if swallowing noise differences between the PET and healthy subjects alone could cause the difference between these groups, we investigated the PeakR in the absence of the nasal click stimulus (Table 3). It was found that there was no statistically significant difference of no-stimulus median PeakR between the Healthy ET and PET groups (*p* = 0.83, Table 4). The difference between median stimulus PeakR and median no-stimulus PeakR for the Healthy ET group was found to be 0.59, and 4.40 for the PET group. Both were deemed to be statistically significant (*p* < 0.001, *p* = 0.031, respectively, Table 5).

**Table 3:** Stimulus and no-stimulus median peak power transmission ratio for healthy ET and PET groups

Healthy ET		PET	
No-stimulus	Stimulus	No-stimulus	Stimulus
0.85	1.44	0.89	5.28

**Table 4:** Wilcoxon rank sum rest for no-stimulus peak power transmission ratio comparison

	Median difference	95% CI	p
Healthy ET vs. PET	0.03	(−0.11 to 0.21)	0.830

**Table 5:** Wilcoxon rank sum test for stimulus and no-stimulus peak power transmission ratio comparison

	Median difference	95% CI	p
Healthy ET Group	0.59	(0.43 to 1.57)	<0.001
PET Group	4.40	(0.46 to 5.73)	0.031

## Discussion

Evaluation of ET function via sonotubometry has been used for many years, but it certainly has its challenges. Primarily, it is difficult to separate out the physiologic noise of swallowing from the transmitted signal noise. This study investigated the acoustic transmission of a newly developed ‘click’ stimulus through healthy ET and PET subjects both without swallowing and during swallow, with the goal of identifying ET opening during swallow and identifying PET. By operating over a wider frequency range, we predict our click stimulus will be more robust in detecting ET events, and less prone to interference from body noise. Of course, the next step would be a side-by-side comparison with traditional single tone sonotubometry, as in principle, more comparison frequencies ought to increase test performance.

Based on our assumption that as the ET is open at rest between swallows in PET, we expected the BaseR would be larger than that of the healthy ET group. Overall, our results agreed with this prediction, as it was found that the median BaseR for the PET group was significantly higher than that of the healthy group, indicating that PET subjects have a lower acoustic impedance at rest than healthy subjects, although there was some variation (Fig. 1).

We hypothesized that when the ET opens in normal subjects, there would be a large increase in amplitude in transmission (PeakR) as the ET is fully opened. In PET subjects, we hypothesized that further opening from swallowing from the resting open state would take it to the same state as the open ET during swallow for healthy subjects, and so expected little difference between PeakR in PET and healthy subjects. Instead, we found a large difference in the peak power transmission in normals versus PET subjects (Fig. 2). In fact, the difference in PeakR is bigger than the difference in BaseR. Three possible explanations are:

1. During swallowing, healthy ETs do not open to the same degree of patency as PETs.
2. During swallowing, the degree of patency in PETs becomes even greater in comparison to the resting state, indicating a fluctuating degree of patency in some subjects.
3. In PET subjects, the physiologic noise of swallowing is transmitted much more than in healthy ET subjects resulting in a higher BaseR.

**Fig. 3:**
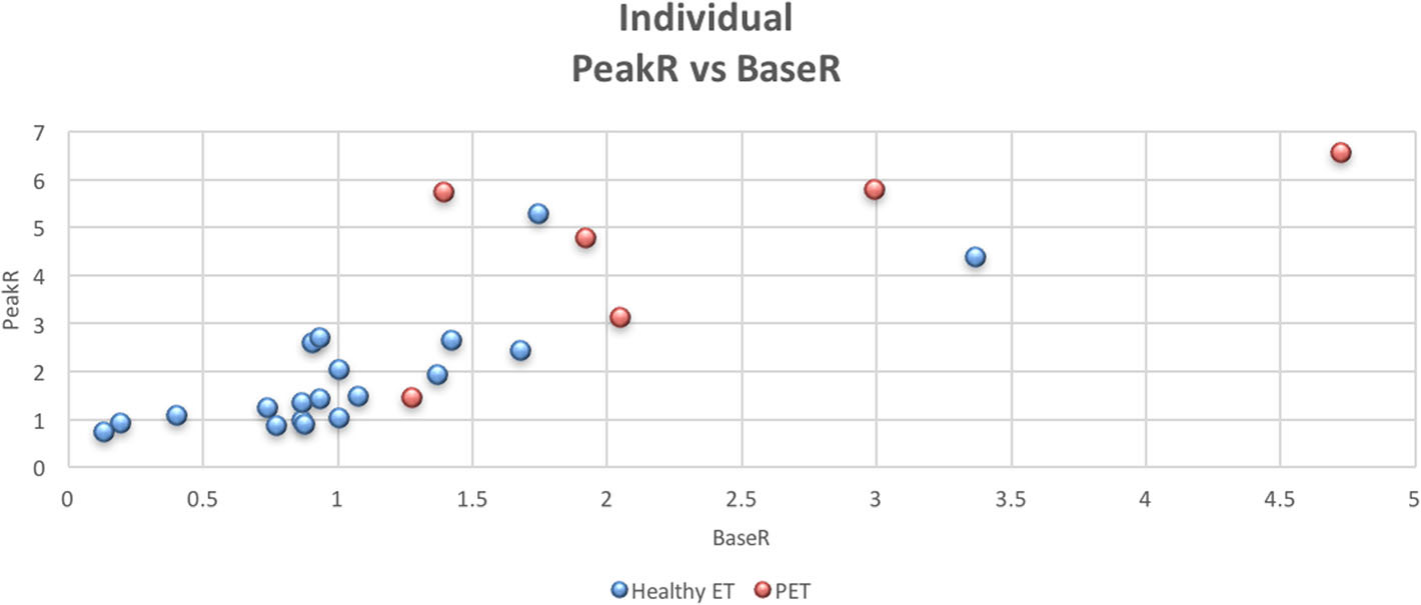
Individual BaseR vs PeakR for healthy ET and PET subjects. A scatter plot comparison of the individual BaseR and PeakR for healthy ET subjects (shown as *blue circles*) and PET subjects (shown as *red circles*). BaseR is represented by the x-axis and PeakR is represented by the y-axis. While there is some variation in both the healthy ET and PET results, the general trend of lower BaseR and PeakR for the healthy group can be observed. This is better appreciated in Fig. 2

However, we examined the latter in the no stimulus condition reported above, and do not find evidence for this. We accept that not every swallow is the same and this method can’t guarantee that the measured swallowing noise is identical to that during stimulus testing. Upon plotting PeakR versus BaseR for the healthy ET and PET patients, a general trend was observed, with healthy ET tending to be in the lower left side of the tracing and PET tending to be in the upper right (Fig. 3). This highlights the two previously mentioned findings: significantly higher BaseR (predicted) and Peak R (unexpected) for the PET group.

The data presented here suggests that PET subjects will yield a power transmission of greater magnitude than healthy ET subjects when tested with a 1000 Hz click stimulus via sonotubometry, both at rest and during swallowing. Our stimulus also seemed able to detect swallowing in normal subjects. However, in the absence of any gold standard for being absolutely certain that there is ET opening during swallowing, it is difficult to be sure that it detects opening and not swallowing noise.

**Fig. 4:**
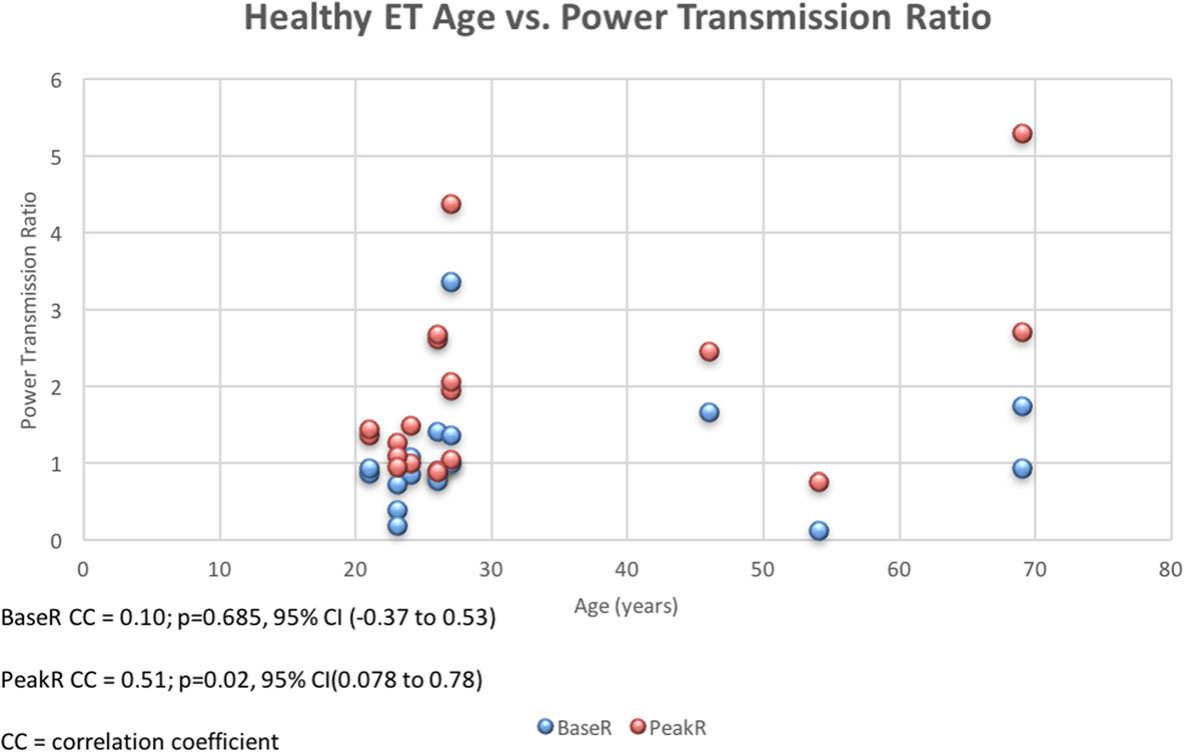
Age vs. PeakR and BaseR for all subjects undergoing sonotubometric testing. A scatter plot of age versus PeakR and BaseR for all study subjects. Age (years) is represented by the x-axis and power transmission ratio is represented by the y-axis. A moderately positive correlation was observed for both BaseR and PeakR, and age (*p* = 0.003, *p* < 0.001, respectively). These findings are highlighted in Table 6

While the healthy ET group mean age was younger than the PET group, one criticism could be that we are not testing PET vs healthy ET, but young vs old ET function. Figure 4 shows a scatterplot between age and BaseR and Peak R in the healthy population. While there is little evidence of a systemic trend for BaseR with age, the was a relationship between age and PeakR (*p* = 0.02, Table 6). However, this was only a moderate correlation and there was no linear relationship observed.

**Table 6:** Summary of correlation testing for BaseR and PeakR, and age for the healthy ET group

	Correlation coefficient	95% CI	p
BaseR vs. age	0.10	(−0.37 to 0.53)	0.685
PeakR vs. age	0.51	(0.08 to 0.78)	0.024

One of the limitations to this study was the sample size in the PET group. However, we wanted to pick only gold standard known PET subjects at the time of testing, and many subjects with intermittent PET did not have eardrum movements at the time of testing, or they stopped between microscopic examination and going to the testing area. It turned out to be surprisingly difficult to find subjects who had continuous movement of the eardrums with respiration, without intermittent loss of this movement even over short time periods. As this is a novel technique being implemented in an uncommon condition, a longer study period may afford a larger population and a closer look into ET function. Comparisons to other types of stimuli are also needed in the longer term.

## Conclusion

To our knowledge, this is the first documented account describing the evaluation of ET function using a 1000 Hz click stimulus. We feel it evaluates the ET over a much wider frequency range than traditional sonotubometry. The evidence presented in this paper suggests that when evaluating ET function via sonotubometric analysis, the novel click stimulus described is a reliable method to determine ET opening in healthy ears, and distinguish between healthy ET and PET states. Further study should be aimed to evaluate this stimulus in a larger population, and to also trial hypo functioning ET patients.

## Abbreviations

BaseR: Baseline power transmission ratio
DAQ: Data acquisition device
EAC: External auditory canal
ET: Eustachian tube
MEC: Middle ear cavity
PeakR: Peak power transmission ratio
PET: Patulous eustachian tube
TM: Tympanic membrane
